# Harry Potter's Occlusion: Report of a Case of Pumpkin Seed Bezoar Rectal Impact

**DOI:** 10.3389/fsurg.2022.902701

**Published:** 2022-07-14

**Authors:** Maurizio Gentile, Lorenzo Vergara, Vincenzo Schiavone, Giovanni Cestaro, Luigi Sivero

**Affiliations:** ^1^ Department of General Surgery, Endocrinology, Orthopedics and Rehabilitation, Federico II University of Naples, Naples, Italy; ^2^ Ospedale di Gallarate ASST Valle Olona, Milan, Italy; ^3^ Department of Medicine and Surgery for Digestive Tract Diseases, Federico II University of Naples, Naples, Italy

**Keywords:** pumpkin seeds, bezoar, occlusion, haematochezia, digital impaction, depression

## Abstract

Bezoar is a term from Arabic “bāzahr” or ultimately from Middle Persian “p'tzhl” (pādzahr, “bezoar antidote” or less commonaly ægagropile or egagropile (2–4). It was believed to have the power of a universal antidote that works against any poison, and a glass containing a bezoar could neutralize any poison poured into it. In science, it is a mass of hair or undigested vegetable matter found in a human or animal intestines, similar to a hairball. Otherwise, the name could derive from a kind of Turkish goat whose name is just bezoar. Usually, it is found trapped in every part of the gastrointestinal system and must be distinguished by pseudobezoar, which is an nondigestible object voluntarily introduced into the digestive tract. The most common causes are a previous gastric surgery such as a gastric band (for weight loss) or gastric bypass, a reduced stomach acid (hypochlorhydria) or decreased stomach size, and a delayed gastric emptying, typically due to diabetes, autoimmune disorders, or mixed connective tissue disease. Seed bezoars are usually found in the rectum of patients without predisposing factors, causing constipation and pain. Rectal impaction is common after ingestion of seeds, while a true occlusion is rare. Although several cases of phytobezoars composed of various types of seeds are reported in the literature, bezoars of pumpkin seeds have rarely been reported. The authors report a case of fecal impaction by pumpkin seed bezoars with abdominal pain: a difficulty to void with subsequent rectal inflammation and hemorrhoid enlargement was observed. The patient underwent a successful manual disimpaction.

## Introduction

In J.K. Rowling's Book of Harry Potter, the apprentice scientist is quizzed on bezoar during the very first Potions Class (1). Bezoar is a term from Arabic “bāzahr” or ultimately from Middle Persian “p'tzhl” (pādzahr, “bezoar antidote”) or less commonly ægagropile or egagropile (2–4). It was believed to have the power of a universal antidote and would work against any poison and that a drinking glass that contained a bezoar could neutralize any poison poured into it. In science, it is a mass of hair or undigested vegetable matter found in a human or animal intestines, similar to a hairball. Otherwise, the name could derive from a kind of Turkish goat whose name is just bezoar.

Usually, it is found trapped in every part of the gastrointestinal system and must be distinguished by pseudobezoar that is an nondigestible object voluntarily introduced into the digestive tract (5, 6).

Bezoars take the name from the core substance so that we can distinguish them: phytobezoars are composed of vegetable fibers and seeds, trichobezoars are formed from hair, lactobezoars are from inspissated milk, and diospyrobezoars are from unripe persimmon fruits (7).

The overall incidence of bezoars is felt to be low and is extremely rare in healthy individuals occurring in far less than 1% of patients in retrospective endoscopic series (7). Kadian et al. (8) reported that they found six cases of gastric bezoars in a 4-year period, during which time 1,400 gastroscopies were done (0.43% of gastroscopies). Ahn et al. (9) reported a similar incidence of 0.43% (14/3,247 esophagogastroduodenoscopy examinations) over a 7-year period. More recently, Mihai et al. (10) noted that there were 49 cases of gastric bezoars over a period of 20 years (0.068% of all endoscopies). Yakan et al. (9) reviewed 432 cases of small bowel obstruction treated within 10 years; of these, 14 (3.2%) cases were caused by phytobezoars. Multiple cases of persimmon phytobezoar (diospyrobezoar) have been reported in regions where the residents frequently consume fresh persimmon fruits and dried persimmons, such as South Korea, Japan, Israel, Spain, Turkey, and the southeastern United States. In a meta-analysis by Ghosheh et al. (11) reviewing 19 reported studies published from 1994 to 2005, laparoscopy was attempted in 1,061 patients presenting with acute small bowel obstruction, and bezoars represented the fifth most common cause, accounting for 0.8% (12).

Certain at-risk groups have been identified and include patients with altered upper GI anatomy after surgery and psychiatric illness or cognitive impairment. The most common causes are a previous gastric surgery such as a gastric band (for weight loss) or gastric bypass, a reduced stomach acid (hypochlorhydria) or decreased stomach size, and a delayed gastric emptying, typically due to diabetes, autoimmune disorders, or mixed connective tissue disease. Other causes are patients who cannot or do not chew their food properly, usually because they have no teeth or poorly fitting dentures and because of an excessive intake of fibers. Edentulous patients with poor mastication of food particles may also be at greater risk for bezoar development, especially if coexisting risk factors, as described above, are also present. In addition, patients with psychiatric illnesses are at an increased risk of bezoar formation due to the possible ingestion of hair and medications (8, 9).

Many cases of bezoars have also been reported in children or adults having psychosocial problems; nevertheless, the condition can occur in normal children with no apparent psychosocial issues (8).

Seed bezoars are usually found in the rectum of patients without predisposing factors, causing constipation and pain. Although the literature has reported several cases of phytobezoars composed of various types of seeds, bezoars formed from pumpkin seeds have rarely been reported (10). The diagnosis may be suggested by the radiologic study and is confirmed by endoscopy. History and digital rectal examination are the mainstays of diagnosis, with manual extraction under local anesthesia being the procedure of choice (13). CT scanning is useful for detecting both gastric and small intestinal bezoars. Phytobezoars are visualized by CT scanning as a round occupational mass in the gastrointestinal tract. Some cases of bezoars can be coincidentally found in asymptomatic patients by esophagogastroduodenoscopy or computed tomography (CT) scanning performed during a health check-up or follow-up of other diseases.

We report a case of a man aged 50 years with a rectal bezoar composed of pumpkin seeds ingested with their shell, necessitating extensive treatment, including manual disimpaction and rectoscopy.

## Methodology

The description of the case follows the 2013 CARE Checklist Guidelines (13).

## Case Report

### Patient Information

A 50-year-old man with no significant medical history was observed in our outpatient room with a 3-day abdominal pain and difficulty passing anything rectally except some sprays of liquid stool. He also complained of hematochezia at defecation. A physical exam revealed a painful tenderness of the abdomen but the presence of normal bowel sounds.

The abdominal wall was mainly painful on the left.

He seemed scared and confused in reporting events and was accompanied by his mother. He reported having spent the Sunday afternoon alone watching TV and eating two bags of pumpkin seeds (about 400 g) with their shell. He was unemployed and showed a depressed attitude. He reported never having had problems of this nature before but having problems of constipation in the last months.

### Diagnostic Findings

**Figure 1:**
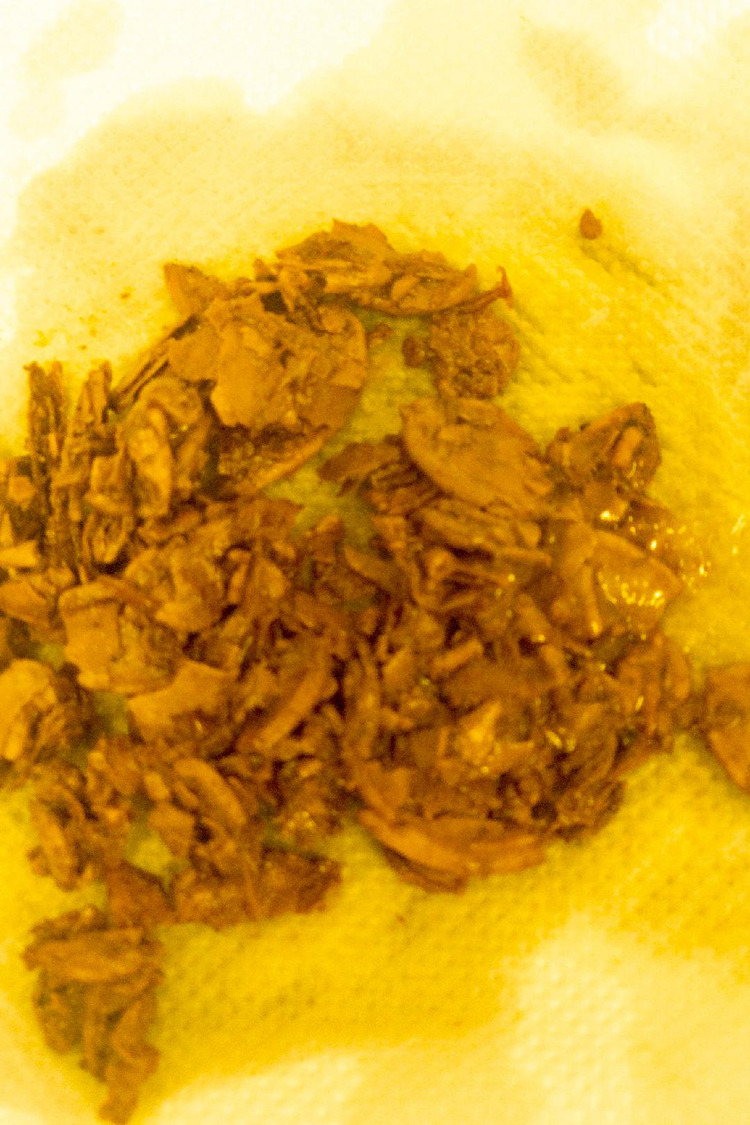
Pumpkin's seeds.

**Figure 2:**
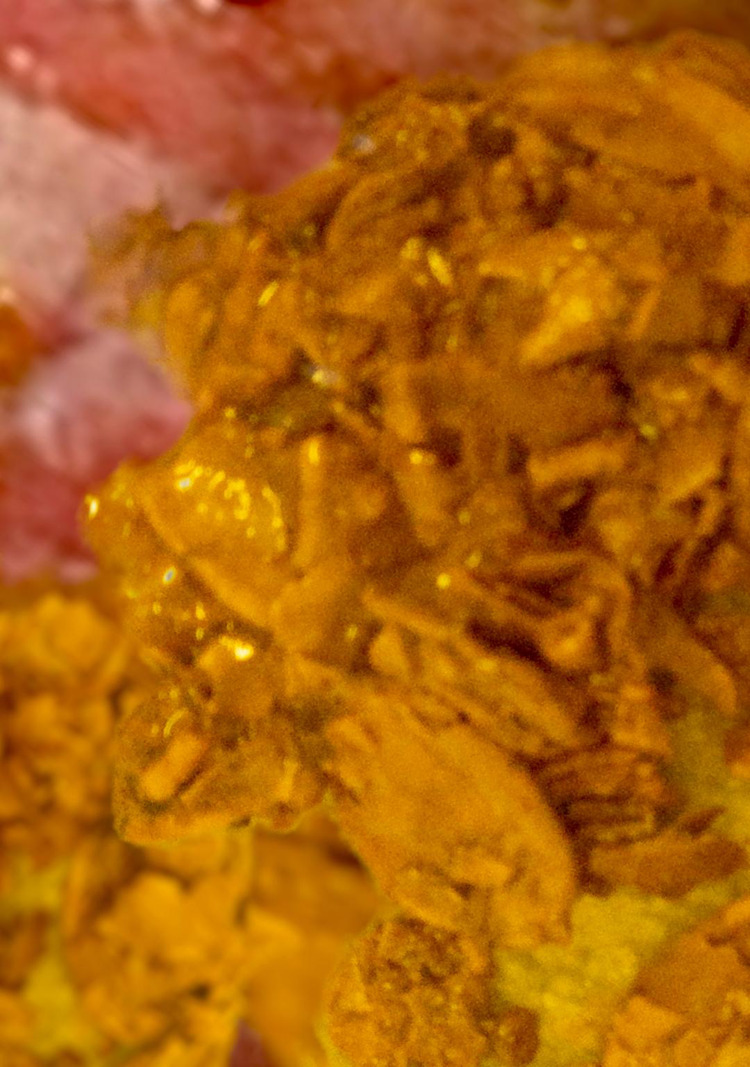
Bezoar in the rectum.

The plain x-ray of the abdomen showed dilatation of the left and bowel. A proctological inspection revealed a hard bolus in the rectum (Figure 1) with blood loss and a rectoscope examination showed a pumpkin bezoar impacted into the rectum (Figure 2). Starting from the history and results of the proctological inspection, the diagnosis of impaction from seeds was quite clear.

### Therapeutic Intervention

Under sedation with propofol, a disimpaction of the bezoar was accomplished with a colon washing. The patient was discharged on the same evening with a prescription of intestinal antibiotics and a large bowel toilet with polyethylene glycol and enemas.

### Outcomes

**Figure 3:**
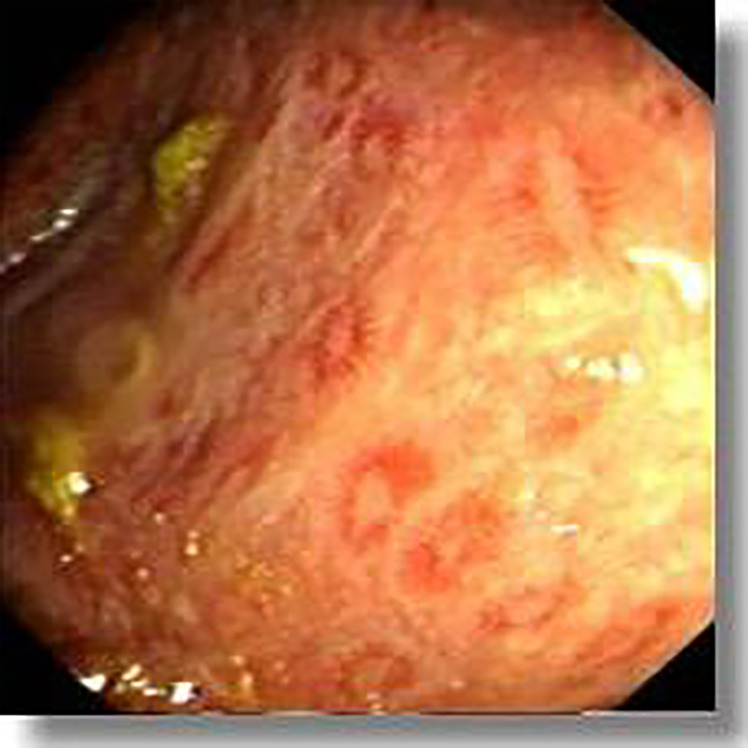
Rectal wall after disimpaction.

Two days after the first admission, the patient returned to the outpatient department complaining of a persistent difficulty to void with a burning sensation and blood loss. A residual hard bolus, smaller than the first, was detected in the rectum, and another disimpaction under local anesthesia was performed. A proctoscopy showed small diffuse ulcerations of the rectal mucosa and enlargement of hemorrhoids (Figure 3). The colon was empty at the end of the procedure. A daily topical application of sucralfate enemas, stool softeners, and fiber diet was prescribed, and the patient passed normal stools without pain the following day. A suggestion of psychological help was made.

### Follow-up

Three weeks after this episode, the rectal mucosa reverted to normal, and the patient declared to move regularly without a burning sensation.

## Discussion

Rectal seed bezoar is an uncommon cause of fecal impaction, more frequent in eastern than western countries and particularly in Middle Eastern and South Asian countries where roasted seeds are very popular (14). The composition of a bezoar is essentially mechanical due to its insoluble and indigestible content. The growth is increased by continuous ingestion of the nondegradable content. The most frequent site is the stomach, and rarely can it be observed in the colon and rectum. Clinical symptoms include nausea, vomiting, anorexia, constipation, and obstipation. Rectal ulcerations are not frequent even if the first report of a stercoral ulceration was described by Berry in 1894: an isolated ulcer produced by pressure necrosis of a fecal mass in the rectum (15).

Seed bezoar is a subcategory of phytobezoar caused by the accumulation of indigestible vegetable or fruit seeds in the intestine lumen. They usually pass the stomach and the ileocecal valve and deposit in the colon up to the rectum, where the compound is dehydrated and forms a hard bolus impossible to evacuate (16). Seed bezoars seem to arise mostly in patients without predisposing factors, as a review from the Manatakis report: 12% of cases of previous gastric surgery, neuropsychiatric illness, and endocrinopathies were reported, contrary to fiber bezoar where rates of risk factors exceed 85% (17).

Seed bezoar occurs most frequently in the rectum both in children and adults, and symptoms are mainly constipation followed by abdominal pain and rectal burning. A true intestinal obstruction is rare, and perforation is reported only in one case (18, 19). Fiber bezoar, due to its location in the stomach, causes specific symptoms such as nausea, vomiting, and abdominal bloating. Manual evacuation under general anesthesia for rectal bezoar is the treatment of choice to avoid discomfort to the patients, while surgery is mandatory in the case of intestinal obstruction from small seeds. Manual disimpaction is the most commonly used procedure both in children and adults, while surgery is more frequent in adults than in children (30% vs. 14.5%). Chemical dissolution of the mass works better with fiber bezoars than with seed bezoars; however, Coca Cola Zero is reported to be effective in breaking a phytobezoar into small pieces (20). Finally, endoscopy is ineffective because, in most cases, the endoscope cannot transit beyond the mass (21). In case of true occlusion, surgery is mandatory even if rare

From 1980 to 2018, 52 studies were reported by Manatakis (16) responding to eligibility criteria over a total of 102 papers published. From 2018 to today, another eight papers with a full text available were published. In four out of the eight, bezoar formation was from seeds (granadilla, mango, and sunflowers in two cases), but none of the patients ate pumpkin seeds (22). According to Manatakis, the major complaint was constipation followed by atypical abdominal or rectal pain. One elderly patient was diagnosed with acute abdomen due to rectal perforation, and one intraoperative incident finding was reported.

Preventive therapy to avoid recurrence must be implemented when the bezoar is removed. The patient should be advised to increase the amount of water intake. Dietary habits must be investigated since inadequate chewing, swallowing whole seeds, or eating seeds with their shell may impact as bezoar (11, 23, 24).

Finally, gastric bezoars are common in cystic fibrosis patients after lung transplantation. The etiology is likely multifactorial, related to gastric motility, respiratory secretions, and medications. Of the 215 patients who received lung transplantation, 17 (7.9%) developed gastric bezoars confirmed by upper endoscopy and 94% of patients with bezoars (16 of 17) had cystic fibrosis (*P *= 0.02), Further investigation is needed to understand the pathogenesis of bezoar formation in this selected population (25).

## Conclusion

Seed bezoar is an uncommon cause of fecal impaction, more frequent in eastern than western countries and particularly in Middle Eastern and South Asian countries. Seed bezoar is a subcategory of phytobezoar caused by the accumulation of indigestible vegetable or fruit seeds in the intestinal lumen: it occurs most frequently in the rectum both in children and adults, and symptoms are constipation followed by abdominal pain and rectal burning. Intestinal obstruction is rare, and perforation is reported only in one case. Manual disimpaction is the commonly used procedure both in children and adults, while surgery is more frequent in adults than in children. Preventive therapy to avoid recurrence must be implemented when the bezoar is removed. An increase in the amount of water intake should be advised. Psychiatric support is mandatory in patients with recurrent episodes of seed ingestion.

## Data Availability

The raw data supporting the conclusions of this article will be made available by the authors, without undue reservation.
